# Une cardiomyopathie révélant un déficit en carnitine palmitoyltransférase I: à propos d’un cas inhabituel

**DOI:** 10.11604/pamj.2020.36.103.23646

**Published:** 2020-06-17

**Authors:** Imane Fetoui, Karima El Fakiri, Noureddine Rada, Ghizlane Draiss, Mohammed Bouskraoui

**Affiliations:** 1 Service de Pédiatrie, Hôpital Mère Enfant CHU Mohammed VI, Marrakech, Maroc

**Keywords:** Cardiomyopathie, carnitine palmitoyl transférase, enfants, *Aeromonas caviae*, Cardiomyopathy, carnitine palmitoyl transférase, children, Aeromonas caviae

## Abstract

Les déficits en carnitine palmitoyltransférase (CPT) sont des atteintes rares due à un défaut d'oxydation des acides gras. Patient de 10 ans sans antécédant présente une dyspnée aigue associée à une toux productive dans un contexte fébrile et d’altération de l’état général. Le patient était polypnéique, tachycarde avec un souffle systolique mitral sans signes d’insuffisance cardiaque. La radiographie thoracique a identifié une cardiomégalie et l'échocardiographie montrait une cardiomyopathie dilatée hypokinétique. Il s’agissait d’un déficit en CPT I. La prise en charge consistait en un traitement de sa cardiopathie et un régime strict hypolipidique et hyperglucidique. Trois mois plutard, le patient se présente une décompensation en insuffisance cardiaque congestive par une infection à *Aeromonas caviae* résistant identifié à l’hémoculture. Une cardiomyopathie dilatée devrait faire penser au déficit en CPT pour une prise en charge précoce qui peut compromettre le pronostic.

## Abstract (en)

Carnitine palmitoyltransferase deficiencies (CPD) are rare and caused by a defect in fatty acid oxidation. We here report the case of a 10-year-old patient with no particular previous history presenting with acute dyspnea associated with productive cough, fever and impaired general condition. The patient was polypneic with tachycardia, mitral systolic murmur and no sign of heart failure. Chest x-ray showed cardiomegaly and echocardiography revealed hypokinetic dilated cardiomyopathy. Carnitine palmitoyltransferase deficiency was diagnosed. Management was based on treatment for heart disease and strict hypopidic and hyperglucidic diet. Three months later, the patient presented with decompensated heart failure due to infection caused by antibiotic-resistant Aeromonas caviae identified in blood culture. CPD should be suspected in patients with dilated cardiomyopathy. This would enable early management which influences prognosis.

## Introduction

Les carnitines palmitoyl transférase sont des substances essentielles pour le transport des acides gras longues chaines du cytosol vers les mitochondries par B-oxydation. Leur déficience peut être à l’origine d’un défaut d’énergie surtout dans les états de crises notamment le stress, les infections ou le jeûne. Le déficit en carnitine palmitoyl transférase (CPT) est l’un des troubles métaboliques de la B-oxydation, faisant suite à une absence ou un manque en CPT I et II. Il est responsable de troubles métaboliques, hépatiques, musculaires et surtout cardiaques. Les enfants atteints vont présenter dès la naissance des cardiomyopathies dilatées ou hypertrophiques souvent mortelles. Nous présentons une observation inhabituelle par son âge d’apparition, son tableau clinique, ses complications et son évolution après un consentement du patient avec une revue de la littérature.

## Patient et observation

Il s’agit de l’enfant A.X, âgé de 10 ans, issu d’un mariage non apparenté avec une notion de mort fœtale in utero (MFIU) chez la mère, une fente palatine opérée à l’âge de 9 mois. Il n’avait pas dans les antécédents desinfections respiratoires à répétition. Ses vaccinations étaient à jour. Le début de son histoire remonte à une semaine par l’installation d’une dyspnée associée à une toux productive et des vomissements alimentaires postprandiaux précédés par des myalgies et une rhinorrhée, le tout évoluant dans un contexte fébrile et d’altération de l’état général asthénie et amaigrissement non chiffré. A l’examen clinique, le patient était conscient, pâle avec des conjonctives décolorées, poids à 28kg (-3 DS) et taille à 123cm (-1 DS), polypnéique à 44 cycles par minute, tachycarde à 126 battements par min. L’examen cardio-vasculaire a noté un souffle systolique mitral sans signes d’insuffisance cardiaque. Par ailleurs, l’examen neuromusculaire et pleuropulmonaire était sans anomalies. Une radiographie thoracique a objectivé une cardiomégalie avec un rapport cardiothoracique à 0,56 sans atteinte du parenchyme pulmonaire et une échocardiographie cardiaque a montré une cardiomyopathie dilatée hypokinétique probablement d’origine virale. Un bilan biologique a identifié une hyperleucocytose à 15940/mm^3^ à prédominance polynucléaires neutrophiles (PNN) à 11580/mm^3^, une protéine C réactive (CRP) à 29,95mg/dl et un bilan inflammatoire élevé avec une VS à 51mm à la première heure. Le patient a été mis sous digital diurétique, IEC et aspirine avec bonne amélioration sous traitement lors son hospitalisation.

Après 6 mois, il y’a eu une réapparition d’une toux chronique persistante compliquée par des épisodes d’hémoptysies de moyenne abondance sans autres signes associés dans un contexte apyrétique et une altération de l’état général persistante. L’examen physique objectivait un patient eupnéique à 22 cycles par minute, tachycarde à 116 battements par minute, normo tendu à 120/50mmHg, avec une bandelette urinaire normale. L’examen cardio-vasculaire montrait un souffle systolique mitral sans autres signes d’insuffisance cardiaque. L’examen pleuropulmonaire a noté des signes de lutte respiratoire avec râles crépitants bilatéraux à l’auscultation. Le diagnostic de tuberculose a été suspecté et le bilan (BK expectorations et Quantiféron) était négatif. Une tomodensitométrie thoracique a montré des foyers de condensation parenchymateuse associés à une cardiomégalie et des signes d’hypertension artérielle pulmonaire (HTAP). Un bilan étiologique immunologique n’a pas objectivé d’anomalies avec des AAN, anti SSA, anti SSB et anti-phospholipides négatives. Un bilan métabolique par dosage de la carnitine a objectivé un taux de carnitine totale et libre élevé à 91,9Umol/L et 53,9Umol/L, acylcarnitine libre abaissé à 0,71Umol/L en faveur d’un déficit en carnitine palmitoyl transférase I. La prise en charge consistait en plus du traitement de sa cardiopathie, en une alimentation avec des repas plus fréquents et en petites quantités pour éviter les hypoglycémies, riches en carbohydrate et une supplémentation en huile de palme.

Trois mois plutard, le patient accuse une orthopnée, un état d’anasarque associant des œdèmes des membres inférieurs, une distension abdominale, une bouffissure du visage et une hydrocèle bilatérale dans un contexte fébrile. Devant la décompensation en insuffisance cardiaque,le patient a nécessité une hospitalisation en milieu de réanimation pédiatrique pendant 2 jours, il a été mis sous VNI et diurétiques de l’anse en seringue auto pousseuse puis transféré dans notre formation après stabilisation pour bilan étiologique. A l’examen, l’enfant étaitictérique, polypnéique à56 cycles par minute, tachycarde à 163 battements par minute, normotendu à 90 /50 mmHg. L’examen cardiovasculaire a noté la persistance du souffle systolique mitral avec un souffle tricuspidien et pulmonaire associé à un éclat de B2 au foyer pulmonaire. Les signes d’insuffisance cardiaque ont été une hépatomégalie douloureuse, un reflux hépato-jugulaire, une turgescence des veines jugulaires et une ascite avec une circulation collatérale de l’abdomen.

L’examen pleuro pulmonaire objectivait un syndrome d’épanchement liquidien bilatéral et la présence de râles crépitants basithoraciques. Une échocardiographie de contrôle a montré une dilatation des 4 cavités avec un cœur gauche hypokinétique et une fraction d’éjection évaluée à 17%. La prise en charge a consisté initialement sur l’augmentation des doses des diurétiques pour atteindre 10mg/kg/J administré en seringue auto-pousseuse et de façon progressive avec une surveillance quotidienne de la tension artérielle et de l’ionogramme sanguin. Le patient a été mis sous régime strict hyposodé, hypolipidique et hyper glucidique ainsi qu’une supplémentation en huile de palme. Par ailleurs, le patient a présenté des pics fébriles lors de son hospitalisation malgré une antibiothérapie par l’amoxicilline acide clavulanique. Des hémocultures réalisées au moment des pics fébriles ont identifié un germe inhabituel. Il s’agissait de *Aeromonas caviae* résistant à l’amoxicilline acide clavulanique et la majorité des céphalosporines de troisième génération. Une antibiothérapie a été instaurée à base de ciprofloxacine pendant 8 jours associée à la gentamycine pendant les 3 premiers jours. L’évolution a été marquée par le gain de l’apyrexieau bout de 72 heures et une amélioration clinique de l’insuffisance cardiaque avec fonte des œdèmes, une régression de l’hydrocèle et une amélioration de la dyspnée. Le contrôle du patient en consultation 1 mois plus tard a montré qu’il était en bonne santé et une échocardiographie cardiaque à intervalle de 1 mois et 3 mois a noté une légère amélioration de la fraction d’éjection passé de 17% à 21%.

## Discussion

Les déficits en carnitine palmitoyl transférase (CPT) constituent les causes les plus répandues des défauts d'oxydation des acides gras (FAO). On distingue: le déficit en CPT I et en CPT II. Le déficit en carnitine palmitoyltransférase (CPT) II en reste le plus fréquent et qui affecte le muscle squelettique. On distingue trois phénotypes de déficit en CPT II: une forme néonatale létale, une forme infantile grave hépato-cardio-musculaire et une forme myopathique légère. La forme néonatale mortelle se présente par une hypoglycémie hypocétosique et des symptômes hépatiques et musculaires sévères. La forme infantile se manifeste aussi par des épisodes d’hypoglycémie hypocétosique pouvant entrainer un coma, associés à un tableau d’insuffisance hépatique aiguë avec hépatomégalie transitoire mais aussi des convulsions [1]. Il existe une forme plus légère, souvent appelée forme de l’adulte, et qui se caractérise par des épisodes récurrents de myalgies, de faiblesse musculaire et de rhabdomyolyse déclenchée lors des exercices prolongés [2]. De prévalence moindre, on trouve le déficit en carnitine palmitoyl transférase I qui lui-même constitué par plusieurs isoformes notamment: hépatique CPT I A, squelettique CPT I B et cérébrale CPT I C, différents par leurs localisations et ainsi par leurs manifestations cliniques [3].

A ce jour, l’enzyme le plus affecté reste celui du foie. L’âge d'apparition se situe entre la naissance et 18 mois mais des cas adultes ont été décrits [4]. Ainsi, on aura des manifestations dès la période néonatale par des épisodes d’hypoglycémies hypocétosiques généralement associé ou non à une insuffisance hépatique aiguë [3]. L'atteinte cardiaque est classiquement absente dans la carence en CPT I mais au cours de ces dernières années, plusieurs cas ont été décrits souvent caractérisés par une cardiomyopathie hypertrophique et plus rarement une cardiomyopathie dilatée, des troubles de rythme ou plus grave une mort subite. Il a été considéré que le défaut d'énergie attribuée par les acides gras longs chaine, source principale d’énergie du myocarde, contribue au développement et à la progression de la cardiomyopathie. En outre, d’autres manifestations ont été décrites notamment l'ischémie et les troubles de reperfusion suite à une accumulation des métabolites toxiques [5]. Ces métabolites seraient également arythmogène et contribuerait à développer une tachycardie ventriculaire polymorphe et une prolongation de l’intervalle QT puisqu’ils altèrent les propriétés des membranes et les protéines intégrales telles que la sarcolemme Na+/K+ ATPase [6].

La carence en CPT1 est presque constamment associée avec un niveau élevé de carnitine plasmatique qui peut donc être un indice très spécifique de la carence en CPT1. Le profil des acylcarnitines plasmatiques et urinaires reste variable mais souvent normal voire abaissé. Dans notre cas, le déficit en carnitine palmitoyl transférase I ne s’est manifesté que par des atteintes cardiaques seules notamment la cardiomyopathie dilatée avec signes d’insuffisance cardiaque congestive. Le patient n’avait ni atteinte neurologique ni atteinte d’ordre hépatique ou métabolique. Tous les bilans biologiques en faveur d’une insuffisance hépatique étaient normaux de même que les glycémies capillaires. Ceci laisse penser que notre patient est probablement atteint d’une carence en variété CPT I B, dite forme squelettique, qui constitue une entité évidemment très rare et peu décrite.

Le traitement reste très limité du fait de l’indisponibilité actuelle d’une supplémentation en enzymes et consiste essentiellement sur la prise en charge des troubles métaboliques. L'hypoglycémie et l'acidose métabolique se corrigent souvent rapidement par un apport approprié, généralement du glucose 10% pour éliminer les métabolites accumulés. Pour le traitement à long terme, il consiste en un régime alimentaire en réduisant l'apport de substrats non métabolisables notamment les lipides. Ainsi, l’alimentation sera basée sur un régime pauvre en graisse et enrichi en triglycérides à chaîne moyenne. De même, éviter le jeûne est également important puisqu’il amène le corps à tenter de puiser ses propres réserves d'énergie sous forme de gras et qui ne peuvent être utilisés. Pendant la petite enfance, le jeûne nocturne au-delà de 6 heures doit être évité avec une alimentation au milieu de la nuit ou de préférence une alimentation continue par sonde. Au-delà de 1 an, une boisson enrichie en amidon ou féculent de maïs au coucher fournit du glucose lent tout au long de la nuit. Certains compléments alimentaires peuvent réduire les antioxydants secondaires et neutraliser les radicaux libres comme la vitamine C, coenzyme Q10 et la vitamine E ou encore la créatinine, l’acide folique et l’acide lipoique qui semblent améliorer la production d’adénosine triphosphate (ATP) [5, 7]. D’un point de vue chirurgical, la transplantation cardiaque n’a pas de place dans les déficits en CPT contrairement aux autres déficits métaboliques. Il est également indispensable de gérer les situations particulières comme les états de stress, l’exercice physique, les chirurgies ou les infections afin d’éviter une décompensation grave comme le cas de notre patient.

L’évolution avait été marquée par une particularité, celle de la bactériémie à *Pseudomonas caviae* qui constitue un germe inhabituel. Les Aeromonas sont des bacilles à Gram négatif, oxydase positive, anaérobies facultatifs. On en distingue 36 espèces, et dont 22 ont été identifiées en clinique depuis 1992. Les plus fréquemment isolées sont, par ordre de fréquence décroissante, *Aeromonas veronii, Aeromonas hydrophila, Aeromonas caviae* et *Aeromonas dhakensis* [8]. Ce sont des germes de l’environnement, ubiquitaires, responsable d’infections parfois très graves et variées telles que les infections cutanées, bactériémies, gastroentérites, péritonites, infections hépatobiliaires, respiratoires ou ophtalmiques. Les portes d’entrée des bactériémies à Aeromonas peuvent être digestives par altération de la muqueuse intestinale ou l’infection des voies biliaires, beaucoup plus rarement, les plaies et cellulites, dont le pronostic est dans ce dernier cas très sombre [9]. Les bactériémies sont poly microbiennes dans 30% des cas. Chez notre patient, la porte d’entrée a été une bactériémie à point de départ respiratoire.

Le genre Aeromonas a la particularité de survenir plus souvent sur un terrain d’immunodépression dont les patients souffrant de pathologie hépatobiliaire ou cancéreux mais aussi de résister aux antibiotiques [10]. Les Aeromonas sont habituellement sensibles aux fluoroquinolones, aminosides, tétracyclines. Ils sont inconstamment sensibles à la tobramycine et au cotrimoxazole. Le traitement repose sur une céphalosporine de troisième génération ou une fluoroquinolone associée ou non à de la gentamicine en cas de contexte grave et le cotrimoxazole en cas de diarrhée nécessitant un traitement antibiotique. Dans notre observation, l’infection par *Aeromonas caviae* s’est révélée sur une hémoculture faisant suite aux pics fébriles chez notre patient. il ne présentait pas de signes digestifs ni cutanés. En effet, pour établir une relation entre le terrain métabolique qu’est le déficit en carnitine palmitoyl transférase I et l’infection. Aucune donnée de littérature, à notre connaissance n’a établi une relation entre les deux entités.

## Conclusion

Le faible taux de survie des enfants atteints de déficit en carnitine palmitoyl transférase suggère que les pédiatres doivent être alertés pour détecter les premières manifestations cliniques dès le bas âge et non pas tardivement quand le pronostic est compromis. La prise en charge reste difficile. A l’heure actuelle, le régime alimentaire reste le pionnier et la seule arme thérapeutique dans nos milieux.
